# Prescribed stimulant medications: Trends in the last decade, pre and post COVID-19 response

**DOI:** 10.1016/j.rcsop.2023.100314

**Published:** 2023-08-09

**Authors:** Meelee L. Kim, Netrali Dalvi, Danielle DeNufrio Valerio, Gail K. Strickler, Leonard D. Young

**Affiliations:** a Institute for Behavioral Health, Heller School for Social Policy and Management, Brandeis University, Waltham, MA, USA; b Office of Prescription Monitoring and Drug Control, Bureau of Health Professional Licensure, Massachusetts Department of Public Health, Boston, MA, USA

**Keywords:** Stimulant prescription drugs, Stimulant medications, Prescription drug monitoring programs, COVID-19 trends

## Abstract

**Background:**

Recent studies indicate that COVID-19 has had a significant impact on access and continuity to opioid and benzodiazepine medications; little is known about its effect on access to and utilization of stimulant medications.

**Objective:**

To investigate trends of dispensed stimulant medications in relation to the COVID-19 pandemic response.

**Methods:**

Stimulant prescriptions dispensed during 2011–2021 were analyzed using the Massachusetts Prescription Drug Monitoring Program (PDMP), the state's data repository for all controlled substance medications dispensed to residents from retail pharmacies and out of state mail-order pharmacies. Statewide trends were estimated by age group, sex, and stimulant-naïve patients (individuals with no stimulant prescription in the prior one-year period).

**Results:**

Overall, stimulant prescriptions increased 70% from 2011 to 2021. Wide differences by sex and age groups were found pre and post COVID response periods. Between 2019 and 2021, stimulant prescriptions for males 12–18 years old decreased 14.6% compared to 0.9% for females. Female stimulant-naïve patients ages 25–34 increased more than males between 2019 and 2021 (11.6% compared to <1%, respectively) and females ages 35–44 increased 4.1% while males decreased by 2.7%.

**Conclusions:**

Administrators, clinicians, and policy makers should closely monitor stimulant prescribing trends, a critical step in improving access to and quality of care.

## 1 Introduction

Stimulant medications are commonly prescribed for attention-deficit/hyperactivity disorder (ADHD) but are also prescribed for other health conditions (e.g., narcolepsy, cognitive impairment, and obesity).^1^^,^^2^ Prescribing of stimulants has notably increased, and studies indicate wide variation of utilization by geography, sex, race/ethnicity, and age in the last decade in the United States.^3^, ^4^, ^5^ Additionally, the increased rate of stimulant prescribing is not concordant with relatively steady rates of new ADHD diagnoses, which suggest increasing off-label use and need for better prescribing guidelines.^3^^,^^6^, ^7^, ^8^ Recent studies indicate that COVID-19 has impacted access to and continuity of certain medications.^3^^,^^9^, ^10^, ^11^, ^12^ However, stimulant dispensing patterns have not received much attention compared to opioid and benzodiazepine medication trends.^13^^,^^14^ This paper explores stimulant prescribing trends in Massachusetts prior to the pandemic to determine a baseline in relation to trends during and just after the statewide COVID-19 pandemic response.

On March 10, 2020, a state of emergency was declared in Massachusetts.^15^ Like most states, public health mandatory requirements and recommendations in response to the spread of COVID-19 evolved throughout most of 2020. For example, all non-essential invasive elective procedures were suspended on March 15, 2020 and a full return to elective procedures did not occur until almost a year afterwards. In parallel, K-12 in-class learning was suspended on March 16, 2020 and did not re-open for the rest of the academic year, and early education centers closed on March 22, 2020 (except for Emergency Child Care Programs) and the Order to allow them to reopen was given on June 26, 2020. While necessary to protect the public, these policies had significant impacts on daily life (e.g., virtual schooling and remote work for non-essential workers) and access to health care (e.g., postponement of elective medical procedures and general treatment).^16^ During the same time, modifications to pharmacy workflows, prescribing methods, and allowable quantities of medications dispensed were made to help ensure continuity in medication access for patients. Monitoring prescribing and dispensation trends following public health crises are important to ensure access to quality care for all. The objective of this paper is to describe trends in stimulant prescription drugs prior to, during, and just after the COVID-19 pandemic response in Massachusetts.

## 2 Methods

The primary data source for this analysis was the Massachusetts Prescription Drug Monitoring Program (Massachusetts PDMP) from 2011 to 2021. PDMPs are electronic databases designed to facilitate the collection, analysis, and reporting of information on the use of controlled substances within a state.^17^ PDMP data offer a unique opportunity (i.e., longitudinal, complete data from community pharmacies) to study controlled substance dispensation trends. The MA PDMP serves as a repository of data for all federally (and specified non-federally) controlled substance medications dispensed at retail pharmacies in MA and from out-of-state mail-order pharmacies. The analytic dataset included prescriptions dispensed to state residents only.

Prescriptions coded as Amphetamines, Amphetamine Derivatives, or Respiratory and Central Nervous System Stimulants in the First Databank Drug file were identified as stimulants for this study. Stimulant records were then aggregated and stratified by the top 3 types of stimulant medications dispensed (amphetamines, methylphenidates, and phentermine), time-period, sex, and age group. Amphetamines and methylphenidates are commonly the first line of medications for ADHD treatment.^18^ Phentermine, a commonly used weight control medication,^19^ was explored for its contribution to the increase in overall stimulant prescribing and the year-on-year trends to determine any anomalies during COVID.

Annual rates (per 1000 state residents) of stimulant prescriptions were calculated by identifying the number of unique patients by stimulant type and dividing by the census estimates for the state by age and sex.^20^ Solid quantity dispensed per patient (SQ/PT) was defined as the count of medications dispensed to a patient in the form of pills, capsules, and tablets. Stimulant-naïve patients, defined as patients who had not received a stimulant prescription in the prior one-year period,^1^ were analyzed to explore disparities in medication access by sex and age. All data were de-identified and program cell suppression rules (*n* < 5) were applied to the final data sets. Since the pandemic response was during 2020, stimulant prescriptions dispensed pre-2020 (pre-pandemic response) was compared to post-2020 (post-pandemic response). Interrupted time series (ITS) analyses were performed using monthly prescription rates per 1000 residents, stratified by age group and sex, with the pre-response period defined as 2018–2019 and the post-response period as 2021–2022. Significant autocorrelations were accounted for using the AUTOREG procedure and statistical significance was based on a *p*-value <.05. All data analyses were performed using analytic software, ProDiver (Dimensional Insight, Burlington MA) and SAS Version 9.4 (SAS Institute, Cary NC).

## 3 Results

**Fig. 1:**
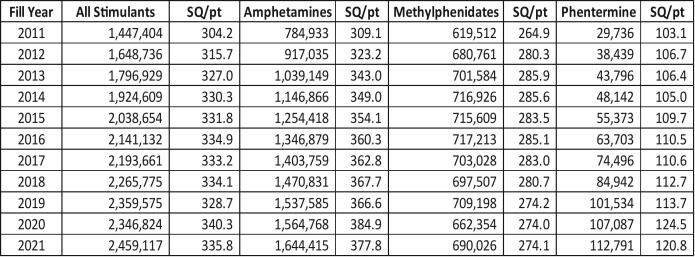
Stimulant Prescription Types by Years - Prescription Counts (Rx) and Mean Solid Quantity per Patient (SQ/pt). Notes: 1) The “All Stimulants” category does not equal the sum of “Amphetamines”, “Methylphenidates” and “Phentermine” categories due to a small number of other stimulant prescriptions (i.e., Phendimetrazine tartrate and diethylpropion HCL) that do not fall into the three main categories of stimulants described. 2) Amphetamines include Amphetamine Sulphate, Benzphetamine HCl, Dextroamphetamine Sulf-Saccharate/ Amphetamine Sulfaspartate, Lisdexamfetamine Dimesylate and Methamphetamine HCl. 3) Methylphenidates include Methylphenidate HCl and Dexmethylphenidate HCl. 4) Phentermine includes Phentermine HCl only.

**Fig. 2:**
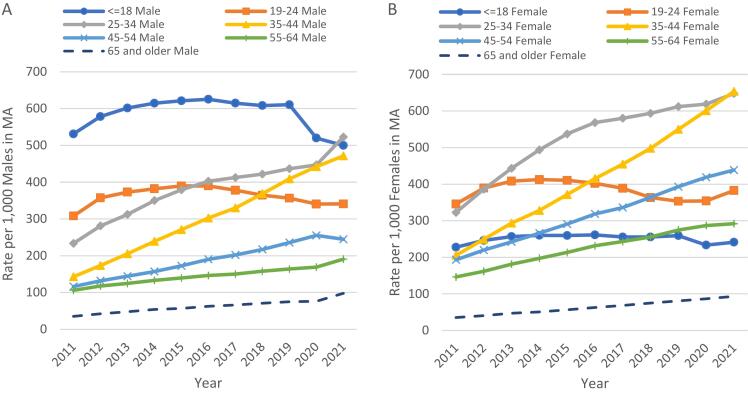
Stimulant Prescription Drug Rates per 1000 Massachusetts Residents by Age Group.

The overall trend from 2011 to 2021, displayed in Fig. 1, show that the annual number of dispensed stimulant prescription drugs to Massachusetts residents increased by nearly 70%. The overall SQ/PT increased by 10% from 2011 to 2021 (304 to 336 pills/tablets, respectively). The SQ/PT for amphetamines increased by 22%, phentermine increased by 17%, and methylphenidates increased by 4%. Fig. 2 displays stimulant prescription rates per 1000 residents across age groups and sex. Between 2011 and 2021, these rates more than doubled for both males and females in ages 25–34 years (234 to 465 prescriptions per 1000 males and 323 to 653 prescriptions per 1000 females). The rates tripled for both males and females in ages 35–44 years during the same timeframe (143 to 478 prescriptions per 1000 males and 206 to 673 prescriptions per 1000 females).

**Fig. 3:**
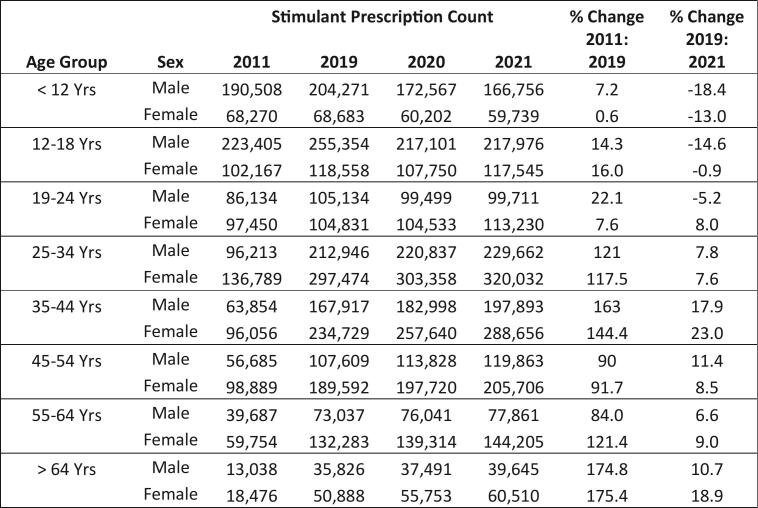
Changes in Stimulant Prescriptions by Sex and Age Group, Massachusetts, 2011 to 2021.

Fig. 3 displays percent change in stimulant prescriptions by sex and age group, over the decade as well as pre-and post-COVID-19 response year. Stimulant prescription counts among patients <12 years decreased across both sexes over the decade. When comparing 2019 to 2021, the prescription count decreased by 18.4% among males and 13% among females, aged <12 years. Among those between 12 and 18 years old, stimulant prescribing decreased by 14.6% for males and 0.9% for females in the same time frame. The largest percent increase across both sexes was seen among ages 35–44 years. ITS analyses revealed similar results in the rate of prescriptions per 1000 population. Both males and females ≤18 years experienced a decline in the level of monthly prescription rate from pre- to post-COVID response year; males had a greater decrease than females (−9.2 and − 1.9 prescriptions per 1000 residents, respectively; *p* < .0001). Females 35–44 years old had a significant level change post-2020 (3.1 prescriptions per 1000 residents, p < .0001) as well as a significant increase in slope relative to pre-2020 (0.2 prescriptions/1000 population per month, p < .0001); there was no significant change in level or slope for males of this age group.

**Fig. 4:**
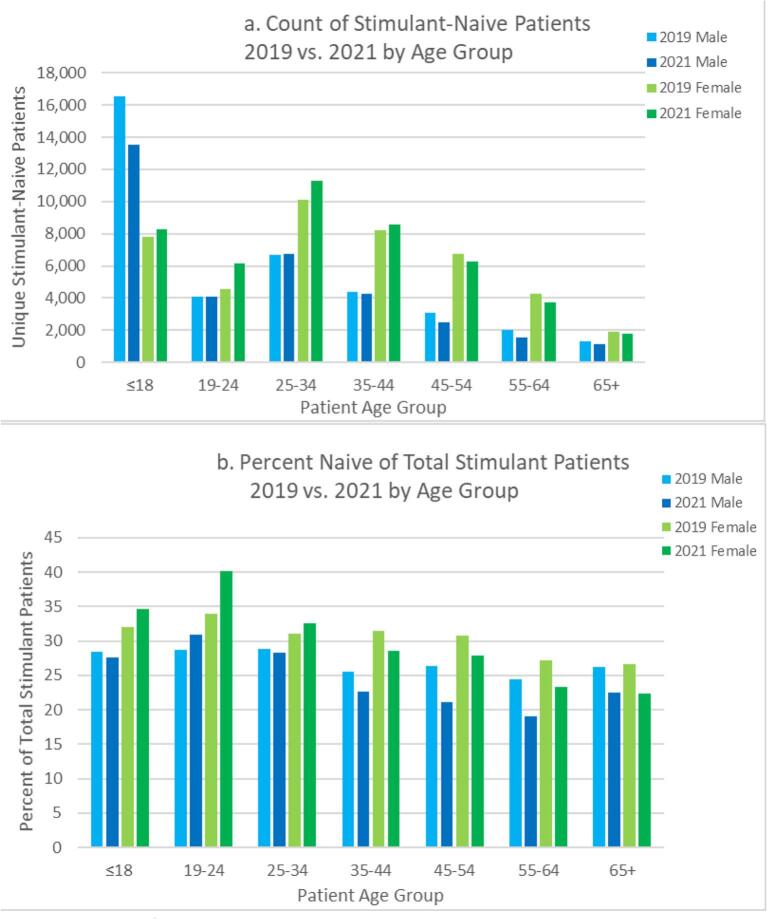
Count and Percent of Naïve-Patients* Receiving Stimulants by Sex and Age Groups from 2019 to 2021. *Naïve-patient defined as individuals who had not received a stimulant prescription in the prior one-year period.

There were differences among stimulant-naïve patients based on age group and sex before and after the COVID-19 response. From 2019 to 2021, the number of school-aged stimulant-naïve female patients (18 years and younger) increased by 5.5% and by 35% for ages 19–24; school-aged male patients decreased by 18.1% and increased by 0.5% for ages 19–24 years. Among stimulant-naïve patients between 25 and 44 years, the change in number of females receiving stimulants was notably different from that of males: females between 25 and 34 years increased by 11.6% compared to <1% for males; females between 35 and 44 years increased by 4.1% compared to a 2.7% decrease for males (see Fig. 4A). The percent of total female patients who were stimulant-naïve increased for all age <35 years but decreased for ages 35 and up; the percent naïve of total male patients increased for ages 19–24 years old but decreased for all other ages groups. The percent of total patients 19–24 years who were stimulant-naïve increased from 33.9% to 40.1% for females and from 28.7% to 30.9% for males (see Fig. 4B).

## 4 Discussion

Changes in dispensation of prescribed medications tied to specific age groups and sex can be considered as pseudo-indicators of broader behavioral health challenges. In the past decade, many states observed increases in stimulant prescribing.^3^, ^4^, ^5^^,^^7^^,^^13^ Analysis of PDMP data also found that there were overall rising rates of stimulants in Massachusetts prior to the pandemic (from 2011 to 2019). However, access to stimulant medications differed by sex, age, and new patient status post-pandemic. For example, stimulant prescribing trend for school-aged youth (18 years and younger) increased between 2011 and 2019 but decreased between 2019 and 2021. The observed seasonal pattern of stimulant dispensation for this age cohort suggests children use stimulants during the school year and often relax or discontinue use when school is not in session.^21^ When virtual learning began in March of 2020, some students may have stopped taking stimulants. Most school districts in Massachusetts returned to partial in-person learning in April 2021 and the 2021–2022 school year resumed in-classroom learning across the state, with schedule adjustments in some districts.^22^ The decline between 2019 (pre-pandemic response) and 2021 further indicates that students did not resume their stimulant prescriptions at the same rate when they returned to the classroom part- or full-time. This trend warrants further exploration and continued monitoring to determine when and if school-aged youth will return to their previous stimulant dispensation patterns.

There were also differences in stimulant prescriptions by sex. Among those in the 19–24 years group, stimulant prescription rates for both males and females followed similar upward trends pre-COVID (2011 to 2019). However, immediately before and after the pandemic response (from 2019 to 2021), rates by sex differed: males continued to decline from 2019 to 2021 while rates for females increased. Among those in the 35–44 years group, stimulant prescribing increased 23%, receiving 1.3 times more stimulant prescriptions compared to males. Although the reasons for the difference by sex is beyond the scope of this study, some studies have pointed to significant impacts on sex inequality and roles during the pandemic.^23^^,^^24^ The pandemic highlighted a gap in work, and labor market choices by sex as work-from-home mandates extended for longer periods of time. Labor statistics point to systemic differences in employment as well as larger numbers of women leaving the workforce. Emerging data explores some shift towards nonegalitarian sex roles spurred by the pandemic, although both sexes cite different sources for their altered mental health. Men mentioned loss of job and economic instability as their primary reason for stress during the pandemic, women more often cited reinforcement of traditional sex roles with disproportionate burden of managing childcare, professional work, and home responsibilities leading to increase in stress and anxiety.^25^ These are noteworthy disparities across age and sex and reasons need to be further examined. For example, although phentermine accounts for a relatively small proportion of overall stimulant prescriptions, females received phentermine at about 5 times the rate than males in 2021 (based on a separate analysis not shown in this paper).

There is a case to be made that the state's requirement for healthcare providers to query the PDMP system for certain controlled substances medications should be extended to stimulants, which also have a high abuse potential and are almost exclusively Schedule II. Some states created dashboards to monitor almost real-time trends in controlled substance prescribing, including stimulants (e.g., Washington).^26^ PDMPs can be a crucial tool to reduce and prevent prescription drug misuse by monitoring dispensation trends. Creating strong surveillance around controlled medications, especially around overlapping prescriptions and multiple provider episodes (i.e., doctor shopping activity), can prove beneficial to policy makers, administrators, providers, and patients. Any new policies should be balanced with appropriate training and guidelines to prevent negative consequences to patients and providers.^27^

This study has some limitations. Race/ethnicity data that may influence stimulant dispensing are not collected by the MA PDMP. Information was not available on prescriptions that were written but not filled, or whether the prescription was used by the intended recipient. The study was unable to determine the appropriateness of the prescription relative to the medical condition. Finally, findings from this study may not be generalizable to other states' trends in relation to their COVID-19 response.

## 5 Conclusions

This study found that COVID-19 had an impact on access to stimulant medications for patients based on age and sex. Monitoring stimulant and other controlled substance medications and overall dispensation trends is critical to improving access to and quality of care, especially during times when public health mandates and recommendations require changes or modifications to standard healthcare practices. Clinicians, administrators and policy makers should work closely with state PDMPs to monitor stimulant prescribing trends by analyzing dispensation data reported to their PDMPs.

## Funding

This work was partially supported by the U.S. Department of Justice, Bureau of Justice Assistance [grant number 2017-PM-BX-0011].

## Declaration of Competing Interest

The Authors declare that they have no conflicts of interest to disclose.

## Footnotes

1 Note, while it is possible that a patient may have received prescription stimulant medications in the past, as long as they have not received a dispensed stimulant medication in the prior one year period they meet the stimulant-naïve inclusion criteria for this analysis.
